# Importance of Micromilieu for Pathophysiologic Mineralocorticoid Receptor Activity—When the Mineralocorticoid Receptor Resides in the Wrong Neighborhood

**DOI:** 10.3390/ijms232012592

**Published:** 2022-10-20

**Authors:** Bruno Griesler, Christin Schuelke, Christian Uhlig, Yekaterina Gadasheva, Claudia Grossmann

**Affiliations:** Julius-Bernstein-Institute of Physiology, Martin Luther University Halle-Wittenberg, 06112 Halle (Saale), Germany

**Keywords:** mineralocorticoid receptor, cardiovascular aging, aldosterone, micromilieu, hypoxia, inflammation, hyperglycemia

## Abstract

The mineralocorticoid receptor (MR) is a member of the steroid receptor family and acts as a ligand-dependent transcription factor. In addition to its classical effects on water and electrolyte balance, its involvement in the pathogenesis of cardiovascular and renal diseases has been the subject of research for several years. The molecular basis of the latter has not been fully elucidated, but an isolated increase in the concentration of the MR ligand aldosterone or MR expression does not suffice to explain long-term pathologic actions of the receptor. Several studies suggest that MR activity and signal transduction are modulated by the surrounding microenvironment, which therefore plays an important role in MR pathophysiological effects. Local changes in micromilieu, including hypoxia, ischemia/reperfusion, inflammation, radical stress, and aberrant salt or glucose concentrations affect MR activation and therefore may influence the probability of unphysiological MR actions. The surrounding micromilieu may modulate genomic MR activity either by causing changes in MR expression or MR activity; for example, by inducing posttranslational modifications of the MR or novel interaction with coregulators, DNA-binding sites, or non-classical pathways. This should be considered when developing treatment options and strategies for prevention of MR-associated diseases.

## 1. Physiologic and Pathophysiologic Role of the MR

The MR is best known for its epithelial functions as part of the renin–angiotensin–aldosterone system (RAAS). The receptor regulates water and sodium reabsorption, mainly in the collecting ducts of the kidney, but also in the sweat glands and colon, to sustain the volume and salt balance of the organism. Proton and potassium excretion are also regulated by epithelial MR [1,2]. However, the MR is also expressed in non-epithelial cells such as neurons, adipocytes, macrophages, fibroblasts, endothelial cells, and vascular smooth muscle cells. Here, MR functions are heterogeneous, and include development and differentiation as well as inflammatory response and stress adaptation [3,4,5]. Next to its important homeostatic function, inappropriate activation of the MR can also lead to pathological effects.

The relevance of MR activity regarding cardiovascular disease has been underlined by studies such as the Randomized Aldactone Evaluation Study (RALES). In this trial, patients with heart failure (NYHA III and IV) received the MR antagonist spironolactone in addition to their standard medication. The addition of the MR inhibitor reduced patient mortality by 30% and hospitalization rate by 35% compared to the control group, which is mainly attributable to the reduced incidence of cardiac complications. Because no change in diuresis or blood pressure was observed in the intervention group compared with patients that received the standard medication, the authors of the study concluded that non-epithelial MR effects contribute to the development and progression of heart failure [6]. The beneficial effect of MR inhibition in patients with heart failure was confirmed by other randomized clinical trials, such as the Eplerenone Post-Acute Myocardial Infarction Heart Failure Efficacy and Survival Study (EPHESUS) and the Eplerenone in Mild Patients Hospitalization and Survival Study in Heart Failure (EMPHASIS-HF) [7,8]. The protective effect of MR antagonist finerenone for the renal function of patients with diabetes mellitus and pre-damaged kidneys has been demonstrated by multi-center studies such as FIDELIO-DKD and FIGARO-DKD [9,10]. Overall, clinical trials indicate that RAAS blockade may prevent the onset of cardiovascular and renal dysfunction and disease [11,12].

The pathologic potential of dysregulated MR activity in the cardiovascular system lies in the induction of inflammation, hypertrophy, and fibrosis, as has been shown in several cell culture and animal experiments [13]. A summary of clinical and preclinical studies examining the effect of MR activation under pathophysiologic conditions can be found in Table 1. 

Oakley et al. could show that the MR plays a pivotal role in stress-induced heart failure using transgenic mice. While cardiomyocyte-specific knockout of the glucocorticoid receptor (GR) induced heart failure in transgenic mice, simultaneous MR-knockout in cardiomyocytes had cardioprotective effects [51]. Inflammation in cardiovascular tissue is sustained and enhanced by the induction of MR target genes such as intercellular adhesion molecule 1 (ICAM-1), a molecule which is expressed by endothelial cells and is relevant for leukocyte diapedesis [52,53]. Excessive MR activation by DOCA and high salt diet in endothelial cells also triggers cardiac inflammation and remodeling by promoting the inflammatory phenotype mediated by vascular cell adhesion molecule 1 (VCAM1) [54]. The MR can also induce the production of reactive oxygen species (ROS) thereby amplifying oxidative stress [55]. Fibrosis induction by the MR is mediated by pro-fibrotic mediators such as connective tissue growth factor (CTGF), transforming growth factor beta (TGF-beta) and galectin-3, which are all regulated by the receptor [11,12]. 

Accumulating data demonstrates that the MR contributes to aging-associated cardiovascular dysfunction, and that MR antagonists benefit the elderly. During aging, alterations of the vasculature precede the clinical development of cardiovascular disease, leading to changes in mechanical and structural properties with losses in arterial elasticity, endothelial dysfunction, and hypertension [56,57]. Local and systemic RAAS work either synergistically or independently to promote pathophysiological effects [58,59]. Aside from inflammation and oxidative stress, altered cell metabolism and induction of a senescence-associated secretory phenotype in adipocytes are the result of pathologic MR activation [32,60,61,62].

The MR in vascular smooth muscle cells (VSMC) of aged arteries shows increased genomic activity and stimulates the expression of proinflammatory cytokines through an epidermal growth factor receptor (EGFR)-dependent mechanism [63]. Cardiac arrhythmias are also frequent complications during aging. It was demonstrated in murine HL-1 atrial myocytes that aldosterone/MR increases the expression of Cacna1h, which may augment intracellular calcium concentrations and facilitate arrhythmias [64].

Today, MR inhibition is an established therapy for heart failure with reduced ejection fraction, but also, for some cases, with preserved ejection fraction. Spironolactone, a first generation MR antagonist, is characterized by relatively low steroid receptor selectivity. It activates the progesterone receptor (PR) and inhibits the androgen receptor (AR) to a significant extent, leading to cycle irregularities in female patients and gynecomastia in male patients [65]. Eplerenone, the second generation MR inhibitor, is much more MR-specific and does not cause PR- or AR-dependent side effects. Still, both compounds affect the renal MR, often leading to hyperkalemia and requiring hospitalization or resulting in treatment discontinuations [66]. While both spironolactone and eplerenone are steroidal MR antagonists, the third generation of MR inhibitors is structurally non-steroidal. From the data available, it can be concluded that finerenone, a representative of the third generation of MR inhibitors, reduces the risk of hyperkalemia while showing comparable efficacy in terms of cardiovascular outcome. This can be explained by the fact that while eplerenone shows approximately three times higher renal than cardiac accumulation, this ratio is approximately balanced for finerenone [67]. It should be noted that this new non-steroidal MR inhibitor also lacks organ specificity and thus, like its predecessors, leads to adverse effects in tissues other than those targeted therapeutically in a dose-dependent manner. For even more potent and specific inhibition of pathologically overactive MR, yet with a flat risk profile, MR signal transduction and the factors that influence it need further investigation [68].

## 2. Over-Active MR in the Absence of Elevated Aldosterone Levels

Questions about the molecular mechanism of pathologic MR activity in cardiovascular disease began to rise when the authors of RALES and EPHESUS announced that the aldosterone plasma levels of the study participants were in the normal range [69,70]. In the 4E-Study, in which Pitt et al. showed that eplerenone is able to effectively reduce left ventricular mass in patients with left ventricular hypertrophy and hypertension, aldosterone levels of the patients were in the normal range as well [71]. Thus, the effects of the MR in the cardio-vasculature seem to depend on additional factors other than enhanced aldosterone levels. This idea is supported by the finding that plasma aldosterone levels are lower in older individuals compared to younger ones [72,73]. Consequently, MR activation may occur in response to aldosterone (aldosterone-dependent activation) or in response to other stimulatory factors (aldosterone-independent activation).

Since aldosterone is not the sole agonist of the MR in the human organism, and glucocorticoids are also able to activate the receptor, glucocorticoid overstimulation of the MR is a hypothesis for excessive MR activity in absence of elevated aldosterone levels [74]. During aging, the activity of 11beta hydroxysteroid dehydrogenase 2 (11beta-HSD2), an enzyme which mediates MR specificity for aldosterone by inactivating cortisol, decreases, making the receptor more vulnerable to glucocorticoids [75]. Inflammatory cytokines are able to increase the expression of 11beta-HSD1 in VSMC. This enzyme catalyzes the opposite reaction to 11beta-HSD2 and increases the concentration of active cortisol. However, 11beta-HSD2 expression in non-epithelial tissues is low, and the cardiac MR is not constantly active [76]. A role for 11beta-HSD2 in salt-sensitive hypertension is discussed elsewhere [77]. Furthermore, it must be noted that the genes induced by the MR upon cortisol-activation in cardiomyocytes differ from those induced upon aldosterone-activation. CTGF and TGF-beta, in this case, are “aldosterone-specific genes” [78]. It also has been shown that the MR does not contribute to cortisol-induced cardiac remodeling in a relevant manner [79]. After all, MR activation by glucocorticoids does not seem to sufficiently explain pathologic MR activity, despite normal aldosterone plasma levels.

The overexpression of the receptor itself could be an explanation for exacerbated MR effects in the absence of elevated aldosterone levels. There is evidence that the susceptibility of aldosterone in spontaneously hypertensive rats is at least partially mediated by a higher expression of the MR in the vascular cells [79]. Cardiac MR expression is elevated in older rats compared to younger ones [32]. MR overexpression has been shown to promote remodeling of ion channels and may promote cardiac arrhythmias associated with heart failure [80].

In addition to altered MR levels in aldosterone target cells, an enhanced activation of the receptor must be considered as a reason for pathologically enhanced MR activity. Many preclinical and clinical studies support the idea of micromilieu changes being a relevant factor in the development of MR-induced pathologies. In the following, it is shown how different micromilieu (affecting) factors influence MR activity.

### 2.1. High Salt Diet

As part of the RAAS, one major function of the MR is the regulation of sodium absorption [1]. In terrestrial vertebrates, most of which tend to have a low sodium intake, the MR is an important protective factor against hyponatremia [81]. Several studies support the hypothesis that excessive sodium intake, which is common in modern societies, leads to pathologically altered MR activity [82,83,84,85]. The fact that sodium deficiency, in contrast, protects against pathological MR activities was shown by an anthropological study on a population in the highlands of New Guinea. There, the inhabitants cover their calorie needs almost exclusively by eating yams. When the minerals of the farmlands are washed out by the monsoon rains, the inhabitants develop a sodium deficiency and, as a result, an increase in serum aldosterone. Despite the elevated aldosterone levels, those under consideration accumulated little cardiovascular disease burden [86]. In the above example, there is a compensatory increase in salt (re)absorption mediated by RAAS activation due to acute salt deficiency. In animal models, it has also been shown that aldosterone-induced remodeling of the myocardium correlates with the sodium intake. Brilla and Weber demonstrated that uninephrectomized rats receiving aldosterone infusions showed myocardial fibrosis only when placed on a high/normal salt diet, but not when deprived of sodium [14]. It should be noted that the salt content of the standard diet is probably already far above the physiological requirements of rodents. Martus et al. were able to show that rats that were able to determine the salt content of their food themselves only consumed about one seventh of the standard diet. The test animals had significantly lower blood pressure compared to the control group. This suggests that even a “normal salt diet” is akin to sodium excess in rats [87]. Mice in which elevated aldosterone levels were induced by gene mutation of the MR target gene Scnn1a showed no significant differences in blood pressure, cardiac fibrosis, or cardiac hypertrophy after 15 months on a normal (0.3%) salt diet compared with normoaldosteronemic control animals [15]. Scnn1 mutation led to renal salt wasting, hyperkalemia, and acidosis due to a loss of function of the epithelial sodium channel (ENaC), which in turn led to pseudohypoaldosteronism type I. This condition is characterized by compensatory activation of the RAAS with elevated renin and aldosterone levels [88]. A high-salt diet induces cardiac remodeling in Dahl salt-sensitive rats. Despite reduced levels of aldosterone in these animals, both eplerenone and esaxerenone (a steroidal and a non-steroidal MR-antagonist) were shown to mitigate cardiac effects induced by high dietary sodium intake [16,17]. In contrast to the salt content of the consumed food, serum sodium concentration seems to have little relevance regarding the effectiveness of MR inhibitor treatment in heart failure [89]. It is important to keep in mind, though, that high salt intake only very seldom leads to hypernatremia, and that serum sodium concentration mostly depends on volume state [90]. Sodium can be stored in the interstitium and endothelial surface layer, where it can contribute to vascular dysfunction and parainflammation through recruitment of inflammatory cells [91,92]. There is clinical evidence that a moderate restriction of salt intake can improve the hospitalization and survival rates of patients with heart failure [93]. However, it must be stated that other studies have led to conflicting results, and that there is a general lack of multi-center studies observing larger study populations [93]. Altogether, these study results gave rise to the hypothesis that only an imbalance in the physiologically inverse relation between salt and aldosterone levels leads to pathologic MR effects in cardiovascular tissues. One assumption of the underlying pathomechanism is that the salt-induced inflammatory micromilieu leads to a change in the redox state of the cells, which then leads to an overactive MR [94].

### 2.2. Hyperglycemia

Increasing evidence supports the role of the RAAS in moding energy balance [95,96]. Hyperglycemia, a condition that frequently occurs in the context of diabetes mellitus, also appears to have a relevant influence on MR activity. Cell culture experiments using human embryonic kidney cells transfected with MR-plasmid (HEK293-MR cells) have shown that high glucose levels upregulate MR expression as well as genomic activity of the receptor [22]. Matrix metalloproteinase-2 (MMP-2), a target gene of the MR which is involved in vascular and cardiac remodeling, shows a higher expression in human cardiac fibroblasts when these are exposed to high glucose levels (compared to normal glucose levels and osmotic controls) [23]. These preclinical findings are supported by a post-hoc analysis of the EPHESUS trial, which found a significantly higher reduction in hospitalization and mortality in diabetic patients with post-acute myocardial infarction heart failure, compared to non-diabetic patients [28]. It is important to note that diabetes mellitus-associated hyperglycemia is also associated with inflammatory tissue responses [97]. An inflammatory cellular milieu also alters MR activity, as described below. Micromilieu changes cannot be considered to be isolated from each other, as they influence or even reinforce each other in many cases.

### 2.3. Inflammation

Several preclinical and clinical studies underline that the MR can be a relevant promotor of inflammation and immune reaction in cardiovascular tissues, partially by the amplification of cytokine release [98]. Pro-inflammatory cytokines themselves seem to be able to enhance MR expression and genomic activity, as has been shown in cell culture experiments with HEK293-MR cells [29]. In the aforementioned study, only costimulation with cytokines and aldosterone, but not each factor on its own, was able to induce inflammation-associated MR target genes such as ICAM-1 and COX2. This implies that the MR depends on prevailing inflammatory conditions to fall into its pathological activity pattern upon aldosterone stimulation [29]. In experiments with mice, a rise in interleukin-1beta (IL-1beta), which occurs after myocardial infarction, led to an amplification of cardiac fibrosis and inflammation, a process which could be suppressed by the use of eplerenone. The use of anakinra, an IL1-receptor antagonist, could reduce inflammation and fibrosis in an equal order of magnitude. This hints at a mutual enhancement of cytokine and receptor activity [30]. IL-1beta stimulation also amplifies the MR-induced expression of MMP-2 in human cardiac fibroblasts [23].

### 2.4. Oxidative Stress

Oxidative stress, elicited by reactive oxygen species (ROS) or reactive nitrogen species (RNS), is another microenvironment factor, which is discussed as an aggravator for pathologic MR activity in the cardiovascular system. ROS evolve endogenously as a side product during cellular respiration in mitochondria, or have a physiological effect during inflammation processes or apoptosis. Reactive nitrogen species such as peroxynitrite can form due to the reaction between NO and superoxide anions. When it was announced that the study populations of the RALES and EPHESUS trials benefited from MR-inhibition regardless of serum aldosterone and sodium concentration, some suspected ROS to be the driver of MR-mediated damage in the cardiovascular system [99]. The idea was that oxidative stress leads to a deficiency in NADH, which is the cosubstrate of 11beta-HSD2. Reduced 11beta-HSD2 activity would, again, permit a higher degree of glucocorticoid-induced MR activation [69]. However, as previously described, there are reasons to doubt that overactivation of the MR by an agonist other than aldosterone is a reason for pathological MR activity in the cardiovascular system. Next to ROS, RNS, which are accumulated in pathologically altered cardiovascular tissues, can also activate the MR. It has been shown in cell culture experiments with HEK293-MR cells that nitrosative stress influenced MR genomic activity. For example, stimulation with the peroxynitrite donor 3-morpholinosydnonimine (SIN-1) activates MR activity, while S-nitroso-N-acetylpenicillamine (SNAP), an NO donor, decreases it. In addition, SIN-1 induces ligand-independent MR translocation from the cytosol to the nucleus. Nuclear translocation only occurred for the MR, while the closely related GR was not affected in its subcellular localization [37]. Aged endothelial cells, especially, can be a source of nitrosative stress. One of the possible mechanisms for this is the uncoupling of endothelial NO synthase (eNOS) through the depletion of tetrahydrobiopterin (BH4), which is an important contributor to endothelial dysfunction [100]. Another pathway of ROS activation is excessive activity of NADPH oxidases, leading to an increase in arterial superoxide levels during aging. This increase in oxidative stress leads to inactivation of nitric oxide, and thereby influences myocardial oxygen consumption in the myocardium of Fischer rats [101]. Aldosterone has been shown to increase superoxide anion formation in macrophages and aorta of mice, and to promote the development of atherosclerotic lesions [102]. Several reports demonstrate that the NADPH oxidase subunits NOX2 and NOX4 can be upregulated in an MR-dependent manner in vascular cells and in cultured neonatal atrial myocytes, and that the MR can thereby modulate its microenvironment [103].

### 2.5. Hypoxia, Ischemia and Reperfusions

Myocardial infarction usually leads to two phases of cardiomyocyte damage: first through hypoxia, and then through reperfusion. This leads to inflammation, oxidative stress, and apoptosis in the cardiac tissues [104]. MR antagonists have proven effective in limiting the ischemia/reperfusion-induced tissue damage and, correspondingly, the infarction zone [105]. Fraccarollo et al. showed that a cardiomyocytespecific deletion of the MR in mice could reduce post-myocardial infarction (post-MI) cardiac remodeling [106]. The group around Bienvenu reported that MR activity aggravates the loss of cardiac function after myocardial infarction in mice, and that this is independent of the application of exogenous MR ligand [107]. Administration of the MR antagonist canrenoate, after induced MI and just before reperfusion, was able to minimize infarct size in a mouse model [108]. The MINIMISE STEMI trial, in which patients were administered a bolus of canrenoate prior to reperfusion followed by a 3-month treatment with spironolactone, did not show a reduction in infarct size compared to the placebo group. MR inhibition could, however, improve the ejection fraction and the left ventricular end diastolic and end systolic volume [49]. Hayashi et. al. also showed that the administration of spironolactone after MI can significantly reduce fibrosis markers [50]. A reduction in mortality and morbidity by MR inhibition in patients who had previously experienced MI was demonstrated by the aforementioned multicenter randomized controlled EPHESUS trial [7]. Taken together, available data suggest that ischemia/reperfusion aggravates pathologic MR activity in the heart, and that the MR takes part in post-MI cardiac remodeling.

In summary, it is likely that aldosterone excess is not the main culprit in most MR mediated cardiovascular pathologies. Two major factors, which account for excessive MR activity, stand out as outlined above: an increased (basal) activation of the MR, and altered MR expression. Both can be induced by microenvironment factors, as described above. In the following sections, the mechanisms by which the microenvironment influences genomic MR activity on a molecular level will be described.

## 3. MR Signaling and Micromilieu

The 984 amino acid-long MR is composed of the N-terminal domain (NTD), the DNA-binding domain (DBD), the hinge region, and the ligand-binding domain (LBD) [1]. The NTD consists of 602 amino acids, and modulates MR activity by interacting with various coregulators. It comprises the activation function 1 and the nuclear localization signal 0 (NLS0) [109]. The DBD is located in the region of amino acids 603–670 and consists of two upright alpha-helices, which interact with zinc ions via cysteine residues to form zinc finger structures. One of the two zinc fingers facilitates binding to the minor groove of the DNA double helix via the “P-box,” and contains a weak nuclear export signal (NES). The other promotes signal transduction-relevant dimerization of the receptor via the so-called D-box [110]. The DBD is followed by the hinge region, which can induce a conformational change in the protein after receptor activation due to its high proline content. This tertiary structural change of the MR leads to interaction of the LBD with the NTD, thus enabling ligand-directed induction of transcription [111]. NLS1, which is composed of several non-directly sequential leucines and determines ligand-independent subcellular localization, can also be found in the hinge region [112]. The C-terminal LBD enables direct interaction with steroid hormones [113]. Additionally, the LBD possesses a ligand-dependent activation function 2 (AF2), as well as the NLS2, which mediates the cytoplasmic-nuclear translocation of the activated MR [114,115]. The AF2 element is an NR-box that contains one or more LxxLL motifs, where L is leucine and x is any amino acid.

Prior to binding to its agonist, the MR in most cells is primarily located in the cytosol [116]. The non-activated MR is conformationally stabilized by chaperone proteins such as heat shock protein 90 (HSP90), HSP70, and different immunophilins [117,118]. Ligand binding triggers the switch between the immunophilins FK506-binding protein 51 (FKBP51) and FKBP52, which allows the MR complex to connect with the dynein–dynactinmotor complex. Once bound to the motor complex, the receptor is translocated into the nucleus together with HSP90 [119]. Here, the receptor dimerizes either with another MR or with a GR [119,120]. Interaction with various coactivators results in chromatin decondensation, receptor dimer attachment to the hormone response element (HRE), and initiation of target gene transcription [94,119]. The MR shares its main HRE with the GR; hence, it is called a glucocorticoid response element (GRE) [94,119]. Although specific mineralocorticoid response elements (MRE), for example, in the promoter region of human EGFR, have been reported sporadically, the physiological and pathophysiological significance of these MRE is largely unknown [121]. The MR-/aldosterone-specific gene expression—despite a shared HRE with the GR—can be explained by differential assembly of MR cofactors [122].

**Figure 1:**
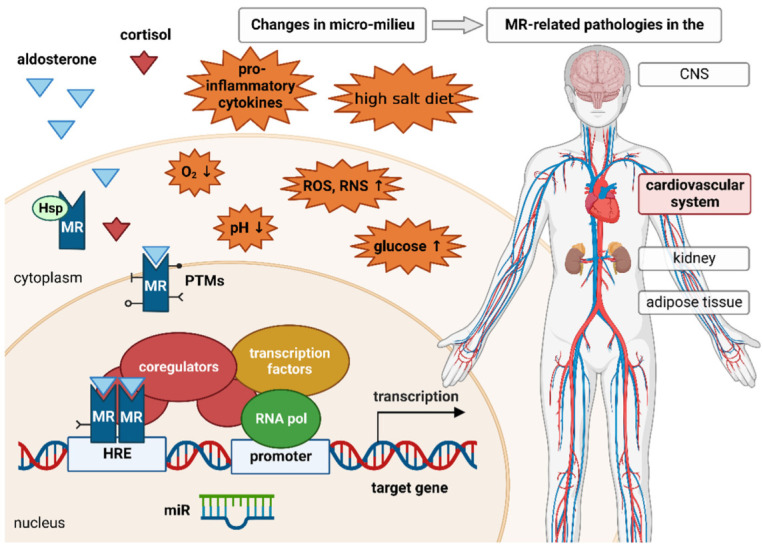
Prior to ligand binding, the mineralocorticoid receptor (MR) is located in the cytoplasm. Its conformation is stabilized by different heat shock proteins (Hsp) and immunophilins. Upon binding of its ligand aldosterone, the receptor is translocated to the nucleus. The MR is also able to bind the glucocorticoid cortisol. In the nucleus, the receptor either homodimerizes with another ligand-bound MR or heterodimerizes with the glucocorticoid receptor (GR) and binds to the hormone response element (HRE), thereby enabling expression of MR target genes. Similarly to other steroid receptors, the MR recruits several coregulators that enhance or repress MR-induced gene expression. MR stability, activity, and signaling are regulated by various posttranslational modifications (PTMs), such as ubiquitination, sumoylation, phosphorylation, acetylation, and oxidation. Micro-RNAs (miR) are able to negatively regulate the expression of the MR as well as several MR target genes. The MR is involved in various pathologies affecting the kidney, central nervous system (CNS), adipose tissue, and, most importantly, the cardiovascular system. These pathologies cannot be exclusively attributed to changes in MR expression or aldosterone concentration. An altered cellular micromilieu due to inflammation, ischemia/hypoxia, oxidative stress, high salt intake, or elevated glucose concentrations can also affect MR signaling. These micromilieu changes can influence the expression of or interaction with several coregulators, miRs, and enzymes responsible for different PTMs, thereby contributing to pathological MR effects.

In addition to this genomic mechanism of action, non-genotropic MR activities have also been described. Most of the non-genomic aldosterone effects are mediated by the classical steroidal MR, located near the plasma membrane and interacting with adjacent molecules [123]. Through interaction of the MR with different cellular mediators, such as protein kinase C-epsilon (PKC-epsilon) or cSrc, the receptor can influence various cellular signaling pathways [124,125]. MR-independent aldosterone effects through the G protein-coupled estrogen receptors GPER have also been demonstrated, but are less well-characterized [123]. In this review, we focus on genomic MR activity, because it is much more comprehensively described than non-genomic MR activity. Modulation of the MR by the micromilieu can occur especially through posttranslational modification, context-dependent coregulator recruitment, and posttranscriptional regulation of MR expression or activity (Figure 1).

### 3.1. Posttranslational Modifications

Posttranslational modifications (PTMs) are structural and functional modifications of a protein catalyzed by specific enzymes. These processes are able to influence the stability, activity, specificity, and signaling of their target proteins, for example, through the fine-tuning of ligand-, coregulator-, and DNA-binding [126]. Micromilieu changes can influence the activity of the modifying enzymes and, thus, affect to what extent posttranslational modifications take place. For the MR, the best investigated modifications are ubiquitination, sumoylation, phosphorylation, acetylation, and oxidation.

Ubiquitination and sumoylation are posttranslational modifications that influence MR protein levels, thereby modulating MR activity and pathophysiological properties. Ubiquitin is a small protein which is activated, conjugated, and, finally, ligated to lysine residues of target proteins by different enzymes (E1-E3). The ATP-dependent reaction is followed by the degradation of the target protein in the proteasome. Under basal conditions, the MR is monoubiquitinated and this PTM is stabilized by the tumor suppressor gene 101 (Tsg 101). This probably contributes to the stability of the unliganded receptor, as it has been shown for the GR [127]. Conversely, binding of aldosterone reduces MR protein levels by inducing phosphorylation events that sever the interaction between Tsg 101 and the MR. This then leads to a polyubiquitination of the receptor, followed by proteasome-mediated degradation [128]. In its basal status in the cytosol, HSP90 protects the MR from polyubiquitination by the ubiquitin-protein-ligase C-terminus of Hsc70-interacting protein (CHIP) and proteasomal degradation [129]. An upregulation of CHIP with a concomitant decrease in MR protein levels could be shown for various types of environmental stress conditions, for example, in HeLa cells that were exposed to oxidative stress [38]. Additionally, there is a link between ubiquitination and phosphorylation when it comes to the regulation of MR protein levels. Protein kinase C-beta (PKC-beta) is activated in cell culture and in mice with elevated glucose levels. PKC-beta activation decreased ubiquitination of MR proteins, thereby protecting the MR from degradation, increasing MR protein levels, and augmenting MR transcriptional activity. Pharmacologic PKC inhibition even lowered the systolic blood pressure in diabetic mice, ameliorating one cardiovascular disease with which MR overexpression is associated [22]. 

Similarly, the addition of SUMO (Small Ubiquitin-like Modifier) to lysine residues of target proteins leads to the degradation of the sumoylated proteins in the proteasome. For the MR, ubiquitin conjugating enzyme 9 (Ubc9) was identified as the conjugating enzyme and protein inhibitor of activated STAT1 (PIAS1) as the ligase [109,130,131]. PIAS overexpression in transiently transfected HeLa cells was followed by MR sumoylation and reduced transcriptional activity upon ligand binding, however, without affecting MR expression [109]. There are indications that PIAS modulates MR activity by sumoylation of cofactors since PIAS overexpression also inhibits MR activity in MR mutants with mutated SUMO-acceptor sites [109,131]. Regarding Ubc9, another ambivalence was illuminated: although Ubc9 is involved in the direct sumoylation process leading to a reduced MR transactivation, Ubc9 is also able to form a complex with steroid receptor coactivator 1 (SRC-1), an MR coactivator, and this complex increases genomic MR activity [130]. Stankovic-Valentin addressed SUMO E1 and E2 as important targets for redox changes caused by ROS [39]. Therefore, there might be a link between sumoylation of the MR and a specific pathological milieu. Nevertheless, further inquiries are needed. Thus, micromilieu factors may affect MR protein levels and activity by modulating posttranslational modifications of the MR or its coregulators. 

Other posttranslational modifications, including phosphorylation, acetylation, and oxidation, modulate MR signaling by influencing the expression or the activity of the receptor. For example, dephosphorylation by protein phosphatase 1-alpha (PP1-alpha) increases MR stability and genomic activity in epithelial cells of the distal tubule, while phosphorylation of MR by ERK1/2 upon aldosterone-binding leads to degradation of the receptor by polyubiquitination, as stated above [132,133]. Galigniana et al. found that MR genomic activity is affected by phosphorylation. They could show that the native rat kidney MR is a phosphoprotein, and that dephosphorylation favors reduced aldosterone binding and increased transformation to the DNA-binding form [134]. The analysis of MR-overexpressing COS-7 cells by mass spectrometry led to the identification of sixteen phosphorylation sites (fourteen in the NTD, one in the hinge region, and one in the LBD) [135]. Different kinases under various cellular contexts induce dissimilar effects by interacting with these phosphorylation sites [136]. For example, phosphorylation of the MR at S843 by Unc-51-like kinase (ULK) in intercalated cells promotes a reduced MR activity and ligand binding affinity. In the case of volume depletion or angiotensin II stimulation, WNK lysine deficient protein kinase 4 (WNK4) inhibits ULK by phosphorylation, resulting in an increased MR activity and affinity [137]. On the other hand, during hyperkalemia, we can find an increased phosphorylation of the MR at S843, which then stimulates potassium excretion. Shibata et al. assume that these opposed reactions play a major role in the fine-tuning of MR-induced gene regulation as a reaction to volume depletion or hyperkalemia [135]. It also shows that MR activity is regulated by factors of the surrounding microenvironment. The phosphorylation of MR-S459 by casein kinase 2 (CK2) seems to increase the transcriptional activity of the MR. Interestingly, CK2 expression, as well as the MR expression, were enhanced in the presence of pro-inflammatory cytokines, as described above. In addition, the MR augmented the CK2-dependent NF-kappaB-signaling, resulting in further expression of pro-inflammatory genes. This mechanism illustrates an interesting link to the correlation between a pathological milieu, augmented PTMs, and the resulting MR function and detrimental effects [29]. The relationship between the MR and the GTPase Ras-related C3 botulinum toxin substrate 1 (Rac1), which belongs to the Rho GTPase family, has been studied intensively in the last years. It has been shown in cell culture, as well as in several animal models, that Rac1 is able to induce nuclear translocation of the MR and MR-dependent gene transcription in the absence of aldosterone [18,19,138,139]. The GTPase is activated by microenvironmental factors such as oxidative stress, elevated glucose concentrations, inflammatory cytokines, and high sodium [20,24,25,31,40]. For some of these activating factors, it has also been shown in cell culture and animal models that they are key drivers of the Rac1-dependent pathologic MR-activation in the presence of relatively low aldosterone concentrations. With the help of Dahl rats, Shibata et al. showed that Rac1 is an essential factor in the development of salt-sensitive hypertension via activation of the MR [19]. Oxidative stress has been shown to activate the MR in a Rac1-dependent manner in rat cardiomyocytes [40]. The molecular mechanism by which Rac1 activates the MR has not yet been elucidated, but it is likely that the GTPase enhances the activity of the MR translocation machinery. It has also been suggested that Rac1 might directly phosphorylate the MR, thereby altering its transcriptional capacities [140]. 

Acetylation of the MR, on the other hand, reduces its transcriptional activity and leads to a diminished expression of MR target genes [141]. Therefore, the balance between lysine acetyltransferases (KAT) and lysine/histone deacetylases (KDAC/HDAC) is of major importance for MR activity. CREB-binding protein (CBP) and p300 were identified as acetyltransferases of the MR, which can also enhance the MR activity as cofactors, depending on the cellular context [142,143]. HDAC3 is the only HDAC for which an interaction with the MR has been described specifically. The knockdown of HDAC3 was followed by increased acetylation and corresponding reduced expression of MR target genes [141]. The same result occurred in reporter gene assays after the utilization of valproic acid, an HDAC-inhibitor. The inhibition of HDAC was even able to attenuate pathological MR effects, such as hypertrophy, hypertension, and fibrosis [141,144]. How the balance between acetylation and deacetylation is regulated and how it might be influenced by a pathological milieu is a subject of current research. For example, there are indications that TGF-beta regulates expression of pathologically relevant genes through p300/CBP and acetylation of histones, as well as SMAD/SP1 in renal mesangial cells under high glucose conditions [26]. Likewise, during inflammation processes, p300/CBP is also utilized by the p65 NF-kappaB subunit to regulate interleukin-6 [145]. 

During the progression of aging and diseases, the production of ROS and RNS increases while the antioxidative capacity of tissues and protective NO concentrations are reduced [146]. In some settings, oxidation of MR impedes binding to ligands as well as binding to DNA, resulting in a diminished target gene expression and reduced biological MR effects [147]. This suggests a new aspect in the development of renal insufficiency in the elderly [148]. The target sites for oxidation are presumably cysteines [149]. Changes in redox state may also facilitate a shift from physiological to pathological MR functions, since different stressors lead to different reactions. For example, NO donors reduce while peroxynitrite donors facilitate MR activity, as has been described above [37].

It must be stated that publications about PTMs of the MR almost exclusively focus on their influence on MR genomic activity. Regarding the influence of PTMs on non-genomic MR activity, the state of data is sparse. It could be speculated that PTMs influencing the protein levels of the MR also affect its non-genomic activity.

### 3.2. Coregulators

Even though the MR binds both aldosterone and cortisol, and also shares a common hormone response element with the GR, the gene expression it induces is ligand-specific [122]. The differential gene expression profile depending on ligand and tissue/cell type is at least partially the result of specific recruitment of coregulatory proteins. Coactivators enable gene expression by facilitating all necessary prerequisites, such as recruiting other proteins necessary for transcription, histone acetylation, unwinding of DNA, chromatin remodeling, initiation of transcription, elongation of RNA chains, RNA splicing, and termination of transcriptional responses [150,151]. Coactivators can also serve as docking platforms for further coregulatory binding to form larger transcriptional complexes [152]. Corepressors have opposite functions to coactivators. Repression of gene expression is usually enabled by subsequent recruitment of histone deacetylase proteins (HDAC). More than 400 coregulatory proteins have been identified for the NR superfamily [153]. Most of them, such as the coactivators SRC-1 and -3, CBP/p300 and PGC-1alpha, and the corepressors NCoR and SMRT, serve as coregulators for several steroid receptors, including the glucocorticoid receptor, the mineralocorticoid receptor, the progesterone receptor, the estrogen receptor, and the androgen receptor. However, some MR-specific coregulators have also emerged that do not interact in the same way with other NRs. Some proteins, such as Ubc9, seem to have dual functions—as corepressors but also as coactivators. The spatial and temporal regulation of this regulatory dualism has not been unraveled yet [130]. The MR structure contains different sites that are able to bind coregulators, termed activation functions (AFs). The LBD is highly conserved among steroid receptors. Therefore, it is not surprising that coregulators that bind to the MR-LBD domain and its AF2 element interact with various other steroid receptors as well. Coregulators specific to MR are usually recruited to the MR-NTD, as the NTD shares less than 15% identity with the other NRs. MR-specific coregulators that have been identified so far include the coactivators eleven-nineteen lysine-rich leukemia (ELL), ubiquitin-like protein SUMO-1 conjugating enzyme (Ubc9), the corepressors protein inhibitor of activated signal transducer and activator of transcription 1 (PIAS1), PIAS3, and nuclear transcription factor Y subunit gamma (NF-YC). Tesmin or metallothionein-like 5 (MLT5) is a ligand-specific MR coactivator that is recruited upon aldosterone binding, but not upon cortisol binding [154]. Other proteins only act as MR coregulators in a specific cellular context, such as Gem (nuclear organelle)-associated protein 4 (Gemin4), eukaryotic elongation factor 1A1 (EEF1A1), or cortisol-specific X-ray repair cross-complementing protein 6 (XRCC6) [154]. Structure-specific recognition protein 1 (SSRP1) even has opposing functions depending on cellular context [155]. Coregulators of MR are comprehensively reviewed by Fuller et al. [142]. Elevated aldosterone levels, MR expression, or MR sensitivity can lead to pathological MR effects, as can dysregulation of coregulatory proteins. One possible reason for changes in coregulator expression or recruitment can be an altered cellular microenvironment due to, e.g., high salt intake, ischemia/hypoxia, inflammation, ROS, RNS, or hyperglycemia. 

Kohata et al. investigated the molecular mechanisms underlying MR-associated salt-sensitive hypertension in a mouse model. They identified the histone demethylase lysine-specific demethylase 1 (LSD1) as a corepressor of MR, and could show that, through this function, LSD1 protects against salt-induced hypertension [156]. Earlier studies found that genetically caused loss of function of LSD1 leads to salt-sensitivity and hypertension in rodents and humans, and also suggested a link between LSD1 deficiency and MR signaling [21]. Additionally, LSD1 plays a vital role in ischemia reperfusion injury, and has been described to demethylate and, thereby, stabilize hypoxia inducible factor-1 alpha (HIF-1alpha) [42]. An upregulation of LSD1 has been described for myocardial, cerebral and renal ischemia reperfusion injury [43,44,45]. Wang et al. claimed that activation of LSD1 in myocardial hypoxic/ischemic injury had cardioprotective effects [44]. Opposed to that, Feng et al. found that upregulation of LSD1 in renal ischemia reperfusion injury results in an increase of oxidative stress by activation of TLR4/NOX4 pathway and accumulation of ROS [43]. During inflammation, LSD1 can act as both a pro-inflammatory and anti-inflammatory epigenetic regulator [42]. LSD1 can have pro-inflammatory effects, and upregulation can contribute to rheumatoid arthritis, sepsis, hepatitis B virus-associated glomerulonephritis (HBV-GN) and SARS-CoV-2 infection, as well as tumor progression [157]. It needs to be clarified which of these effects are mediated by MR, as LSD1 acts as coregulator for several NRs. 

ELL (eleven-nineteen lysine-rich leukemia) is a coactivator of MR that discriminates between MR and GR genomic signaling, as it also acts as a corepressor of GR while having no effect on the other steroid receptors [158]. Under oxidative stress, glucocorticoids can promote cardiac fibrosis via MR and its coactivator, ELL. Treatment of rat cardiac fibroblasts with corticosterone and H_2_O_2_ led to fibrosis, upregulation of ELL expression, and binding of ELL to the MR. The effects were attenuated by MR antagonist eplerenone or knockdown of ELL. Therefore, ELL might be an important target for the treatment of cardiac fibrosis [41]. ELL also plays a role in hypoxia response in cancer cells, where HIF-1alpha and ELL seem to modulate each other’s function [46]. ELL-dependent regulation of the MR under hypoxic conditions has not yet been described.

PGC-1alpha is a coactivator of MR and other steroid receptors, whose expression is strongly influenced by the cellular microenvironment. Decreased PGC-1alpha protein levels have been found in the cardiac tissue of aged rats [32,159,160]. TNF-alpha, TWEAK, (TNF-like weak inducer of apoptosis) and interleukin 1, known to be mediators of inflammation, reduce the expression of PGC-1alpha in the skeletal muscle, liver, and kidney. [33,34,35]. NO regulates PGC-1alpha expression in endothelial cells differentially. While short-term (<12 h) exposure of endothelial cells to NO leads to an increase in PGC-1alpha protein levels, long-term (>24 h) exposure leads to a downregulation of the coregulator [161]. A decreased level of PGC-1alpha is linked to mitochondrial dysfunction and increased oxidative stress. This effect is mediated by MR, as the MR antagonist eplerenone partially blocks the negative effects of oxidative stress on mitochondrial integrity [32]. Therefore, an increase in MR expression or activity may contribute to age-related cardiac dysfunction or other diseases, partially by decreasing mitochondrial renewal mediated by PGC-1alpha, impairing mitochondrial ultrastructure, and inducing ROS accumulation. PGC-1alpha dysregulation also plays a role in kidney and liver disease, as well as diabetes and pancreatitis [162]. An inflammatory microenvironment, and especially TNF-alpha and interleukin 1, can also affect the expression of the steroid receptor coactivators 1 and 2 (SRC-1 and SRC-2), which regulate all steroid receptors [34]. Mutations of other steroid receptors can also lead to altered binding and recruitment of cofactors. Morales et al. observed that the use of AR inhibitors caused heart failure and myocarditis in a patient carrying a mutation in the MR-NTD adjacent to the AF1a site [159]. They claimed that AR inhibition caused MR activation due to altered binding of coregulators. This highlights the importance of balanced overall steroid receptor signaling. In summary, micromilieu changes can affect general cofactors of the steroid receptor family, but also some cofactors specific for the MR, and may therefore help explain MR specificity and pathological MR effects.

### 3.3. Non-Coding RNAs

In recent years, non-coding RNAs and, of these, especially micro-RNAs (miR) have been studied intensively in their role as relevant regulators of cellular and inter-cellular processes [163,164]. MiRs act as negative regulators of protein expression by suppressing the translation of their complementary mRNA and/or inducing their degradation [165]. In order to enfold their activity, these approximately 21-nucleotide-long RNA molecules interact with different proteins (most importantly of the Argonaute family) to form the miR-induced silencing complex (miRISC) [166,167,168]. Since posttranscriptional regulation of gene expression by miRs is very common, it is not surprising that MR signaling is subject to this regulation as well. MiRs modulate MR signaling by regulating the expression of the receptor itself, but also by altering the expression of MR target genes [169]. Other modulators of MR activity, such as 11beta-HSD2, are also known subjects of miR-bound regulation [170]. For example, the miR-466 family, consisting of mir-466a, miR-466b, miR-466c, and miR-466e, not only negatively regulates the expression of the MR, but also the expression of one of its well-known target genes, SGK1. It could be shown that the expression of the miR-466 family is induced by aldosterone in the collecting duct, suggesting a negative feedback loop [171]. While some of the miRs seem to be part of negative feedback loops in MR regulation, there is also evidence that, under pathologic conditions, the expression of MR regulating miRs decreases. It is likely that the lack of MR silencing or degradation is relevant to the progression of MR-mediated pathologic processes. The first miRs that were identified as suppressors of MR expression were miR-124 and miR-135a [172]. The effect of reducing MR protein levels by binding to the 3′-untranslated region of MR-mRNA was confirmed by luciferase reporter assay [173]. The long non-coding RNA MALAT1, which is associated with hypertension and other detrimental cardiovascular effects, has been shown to elevate endothelial MR levels via sponging miR-124 and miR-135a [174,175]. This process can be modulated by environmental factors. With the help of an animal model, Mannironi et al. have shown that acute stress induced by restraining mice was able to downregulate miR-124 and miR-135a, and thereby upregulate MR expression in neuronal cells of the amygdala [173]. In septic mice, the myocardial upregulation of HDAC1 led to lowered miR-124 levels, resulting in greater myocardial injury [176]. In hepatocellular carcinoma cells, miR-766-3p has been shown to reduce MR expression and, thereby, promote proliferation and metastasis [177]. Hayakawa et al. could show that this miR is also involved in the containment of synovial inflammation by inhibiting NF-kappaB activation. It has also been determined that NF-kappaB inhibition by miR-766-3p was at least partially depending on the suppression of MR expression [178].

Another inflammation-associated miR that downregulates the MR is miR-155-5p. Reduced blood levels of miR-155-5p were not only found in patients with coronary artery disease with intermittent hypoxic states for cardiomyocytes, but also in patients with diabetes mellitus and ischemic heart disease [179,180,181]. Additionally, age was inversely correlated to miR-155-5p blood levels [179]. High MR abundance suppresses miR-155 levels, and thereby increases L-type calcium channel (LTCC) and angiotensin-II receptor-I expression, leading to oxidative stress, vasoconstriction, and hypertension. In contrast with these results, Marques et al. found elevated levels of miR-155-5p in the plasma of patients with heart failure, casting some doubt about miR-155-5p being a cardio-protective micro-RNA [182].

Moreover, inflammation, hypoxia and ischemia/reperfusion also influence miR regulation of MR signaling. In neuronal cells, SOX2 overlapping transcript (SOX2OT), a long non-coding RNA, is upregulated upon ischemia/reperfusion simulation. This long non-coding RNA sponges miR-135a, thereby upregulating MR expression. A similar mechanism is conceivable in cardiomyocytes [47]. The same applies to the long non-coding RNA Gm11974, which inhibits the effect of miR-766-3p on MR expression. Like SOX2OT, Gm11974 is upregulated in neuronal cells exposed to ischemia/reperfusion stress [48]. In cardiomyocytes, miR-181a seems to attenuate post-myocardial infarction tissue remodeling promoted by the MR, at least partially by suppressing A disintegrin and metalloproteinase with thrombospondin motifs 1 (ADAMTS1), a target gene of the MR. This was reported by Garg et al. based on cell culture and mouse studies [183]. MiR-181a also displays anti-atherosclerotic properties by inhibiting NF-kappaB activation in the vascular endothelium [184]. NF-kappaB is an important down-stream signaling molecule of aldosterone-MR-signaling [185]. In hypertrophic rat myocardial tissue, miR-181a expression is decreased, resulting in excessive angiotensin II-induced autophagy and, thereby, aggravated heart remodeling [186]. Overall, inflammatory processes, hypoxia, or ischemia/reperfusion have a high impact on miRs regulating MR signaling.

Other micromilieu factors that affect miR signaling are hyperglycemia and oxidative stress. LINC-P21, a long non-coding RNA known to be upregulated in patients with type 2 diabetes mellitus (T2D), has been shown to inhibit the expression of miR-766-3p, thereby upregulating MR expression in pancreatic islet cells. This process is assumed to be relevant for the progression of beta-cell dysfunction in T2D [27]. Guo et al. could show that miR-454 is also able to reduce MR expression in oral squamous carcinoma cells [187]. In patients with heart failure, miR-454 is significantly lowered compared to healthy controls. Treatment of rat cardiac H9c2 cells with H_2_O_2_, mimicking oxidative stress, resulted in reduced miR-454 levels as well [188]. 

## 4. Conclusions

Inhibition of the MR has become an integral part of heart failure therapy [189]. While spironolactone, as one of the first MR inhibitors, carried a wide range of side effects, its successors became increasingly tolerable due to an increase in selectivity [190]. Nevertheless, additional ways to modulate RAAS activity are of interest to further attenuate pathological cardiovascular changes. In recent years, the aldosterone synthase CYP11B2 has been investigated as a potential therapeutic target for the treatment of different cardiovascular diseases. CYP11B2-inhibition could be an innovative strategy to treat illnesses accompanied by high aldosterone concentrations, such as drug-resistant hypertension, on the basis of primary hyperaldosteronism. As outlined above, however, their efficacy is unlikely to provide much additional benefit for the majority of cardiovascular patients, because factors other than high aldosterone concentrations seem to play a major role in pathophysiologic changes. Therefore, we hypothesize that one additional approach to further minimize MR activation may be to modulate the pathological microenvironment or the factors activated by it. The pharmacological suppression of Rac1 activity to attenuate pathologically active MR is a therapeutic approach which has been proposed by some authors [191,192]. Different small molecule inhibitors of Rac1 have been designed, but there has not been a clinical testing of any of these substances yet. Since Rac1 is likely to be one of the most relevant mediators of the effects the pathologic microenvironment has on the MR, its direct inhibition is a tempting idea. Statins, which are renowned for their cardioprotective effects, are also known for their capacity for Rac1 inhibition [193]. Due to their pleiotropic effects, however, it seems necessary to develop tolerable direct inhibitors of Rac1 [192]. It must be noted that, apart from Rac1, the majority of micromilieu conditions and their mediators are modulators of MR activity and are not capable of activating the MR in absence of any MR ligand [18,19,138,139]. This implies that aldosterone is still an important factor in MR-associated pathologies. Aldosterone significantly increases the cytokine-induced expression of pro-inflammatory genes such as cyclooxygenase-2 (COX2) via MR, underlining its importance in MR-mediated inflammation [29]. Patients suffering from primary hyperaldosteronism more frequently experience cardiovascular events, including myocardial infarction and stroke [191]. Furuzono et al. could show that pharmacologic inhibition of aldosterone synthase reduces mortality in mice with pressure-overload-induced heart failure [194]. It will be interesting to compare the efficacy of aldosterone synthase inhibitors and MR antagonists in the treatment of cardiovascular and renal diseases. 

The GTPase Rac1, however, is not only of interest when it comes to pathologies of the cardiovascular system, but is also known to promote cancer chemoresistance, radioresistance, and immune evasion. Therefore, it is a molecular target that is also intensively studied in oncology research [195]. Other known modulators of MR activity, such as CK2, are promising targets for anti-cancer therapy as well [196]. As cardioprotection becomes more and more relevant in chemotherapy protocols, these interconnections should be investigated even more intensively in the future [197]. Overall, MR signaling is subject to many intra- and extracellular influences. The investigation of how micromilieu changes affect MR signaling on a molecular basis seems promising for the development of therapeutic strategies to tackle MR pathologies.

## Figures and Tables

**Table 1:** Preclinical and clinical studies evaluating the role of micromilieu changes for the development of MR-related pathologies.

Micromilieu Change	Effect on MR	Reference
*high salt diet*	**Pre-clinical findings**	
	Elevated aldosterone levels cause cardiac fibrosis only on high/normal salt diet but not when deprived of sodium in rats and mice.	[14,15]
	MR antagonists mitigate high-salt diet-induced cardiac remodeling in Dahl salt-sensitive rats.	[16,17]
	The GTPase Rac1 is able to induce nuclear translocation of the MR and MR-dependent gene transcription in the absence of aldosterone in salt-induced kidney injury.	[18,19]
	Rac1 mediates NaCl-induced superoxide generation.	[20]
	Dysregulation of the MR corepressor LSD1 leads to salt-sensitivity and hypertension in rodents and humans.	[21]
*hyperglycemia*	**Pre-clinical findings**	
	High glucose levels upregulate MR expression and MR transcriptional activity in cell culture experiments.	[22,23]
	High glucose levels activate kinase PKC-beta, promoting MR expression and transcriptional activity and reducing ubiquitination in mice.	[22]
	High glucose induces Rac1 activation in mesangial cell culture.	[24]
	Rac1 is required for cardiomyocyte apoptosis during hyperglycemia.	[25]
	TGF-beta1 induces expression of pathological genes involved in diabetic nephropathy via CBP (CREB-binding protein)/p300, a histone acetyltransferase and coactivator of the MR, in rat renal mesangial cells in high glucose conditions and in glomeruli from diabetic mice.	[26]
	LINC-P21, a long non-coding RNA known to be upregulated in patients with type 2 diabetes mellitus, upregulates MR expression in pancreatic islet cells.	[27]
	**Clinical findings**	
	The use of MR antagonist eplerenone leads to a significant reduction in instances of hospitalization and mortality in diabetic patients with post-acute myocardial infarction heart failure compared to non-diabetic patients.	[28]
*inflammation*	**Pre-clinical findings**	
	Costimulation of MR with cytokines and aldosterone induces the expression of inflammation-associated MR target genes.	[29]
	The expression of MR and CK2, a kinase phosphorylating the MR, thereby increasing its transcriptional activity, is enhanced in the presence of pro-inflammatory cytokines.	[29]
	IL-1beta influences MR signaling amplifying cardiac fibrosis in mice.	[30]
	TNF-alpha stimulates Rac1 activity.	[31]
	TNF-alpha, TWEAK, and interleukin 1 reduce the expression of the MR coactivator PGC-1alpha, which is associated with mitochondrial dysfunction and increased ROS accumulation.	[32,33,34,35]
	TNF-alpha and interleukin 1 affect the expression of MR coactivators SRC-1 and SRC-2.	[34,36]
*oxidative stress*	**Pre-clinical findings**	
	RNS accumulation in pathologically altered cardiovascular tissues can activate the MR by promoting its nuclear translocation.	[37]
	Upregulation of ubiquitin-protein-ligase CHIP marks the MR for proteasomal degradation under oxidative stress in HeLa cells.	[38]
	SUMO E1 and E2 enzymes that also mark the MR for sumoylation and degradation are affected by redox changes caused by ROS.	[39]
	Oxidative stress causes MR activation in rat cardiomyocytes via Rac1.	[40]
	Under oxidative stress, glucocorticoids can promote cardiac fibrosis through MR via upregulated expression of its coactivator ELL and binding of ELL to the MR in rat cardiac fibroblasts.	[41]
*hypoxia, ischemia and reperfusion*	**Pre-clinical findings**	
	Dysregulation of the MR corepressor LSD1 plays a role in myocardial, cerebral, and renal ischemia reperfusion injury by stabilizing HIF-1alpha.	[42,43,44,45]
	MR coactivator ELL and HIF-1alpha modulate each other’s function during hypoxia response in cancer cells.	[46]
	In neuronal cells, SOX2OT and Gm11974, long non-coding RNAs that upregulate MR expression, are upregulated upon ischemia/reperfusion.	[47,48]
	**Clinical findings**	
	Post-MI inhibition with eplerenone reduces mortality and morbidity.	[7]
	Peri- and post-reperfusion MR inhibition can improve the ejection fraction and the left ventricular end diastolic and end systolic volume.	[49]
	Administration of spinolactone after MI can significantly reduce fibrosis markers.	[50]
